# Plumbagin alleviates temporomandibular joint osteoarthritis progression by inhibiting chondrocyte ferroptosis via the MAPK signaling pathways

**DOI:** 10.18632/aging.205253

**Published:** 2023-11-29

**Authors:** Tiehan Cui, Yun Lan, Fei Yu, Suai Lin, Jiaxuan Qiu

**Affiliations:** 1 Department of Oral and Maxillofacial Surgery, The First Affiliated Hospital of Nanchang University, Nanchang 330006, China; 2 Department of Stomatology, Beijing Hospital of Integrated Traditional Chinese and Western Medicine, Beijing 100039, China; 3 Medical Innovation Center, The First Affiliated Hospital of Nanchang University, Nanchang 330006, China

**Keywords:** temporomandibular joint osteoarthritis, plumbagin, ferroptosis, MAPK pathways

## Abstract

Aims: The acceleration of osteoarthritis (OA) development by chondrocytes undergoing ferroptosis has been observed. Plumbagin (PLB), known for its potent antioxidant and anti-inflammatory properties, has demonstrated promising potential in the treatment of OA. However, it remains unclear whether PLB can impede the progression of temporomandibular joint osteoarthritis (TMJOA) through the regulation of ferroptosis. The study aims to investigate the impact of ferroptosis on TMJOA and assess the ability of PLB to modulate the inhibitory effects of ferroptosis on TMJOA.

Materials and methods: The study utilized an *in vivo* rat model of unilateral anterior crossbite (UAC)-induced TMJOA and an *in vitro* study of chondrocytes exposed to H_2_O_2_ to create an OA microenvironment. Various experiments including cell viability assessment, quantitative RT-PCR, western blot analysis, histology, and immunofluorescence were conducted to examine the impact of ferroptosis on TMJOA and evaluate the potential of PLB to mitigate the inhibitory effects of ferroptosis on TMJOA. Additionally, RNA-seq and bioinformatics analysis were performed to investigate the underlying mechanism by which PLB regulates ferroptosis in TMJOA.

Results: Fer-1 demonstrated its potential in mitigating the advancement of TMJOA through its inhibitory effects on ferroptosis and matrix degradation in chondrocytes, thereby substantiating the role of ferroptosis in the pathogenesis of TMJOA. Furthermore, the observed protective impact of PLB on cartilage implied that PLB can modulate the inhibition of ferroptosis in TMJOA by regulating the MAPK signaling pathways.

Conclusions: PLB alleviates TMJOA progression by suppressing chondrocyte ferroptosis via MAPK pathways, indicating PLB to be a potential therapeutic strategy for TMJOA.

## INTRODUCTION

Temporomandibular joint osteoarthritis (TMJOA) is a widespread and persistent joint disease afflicts millions of people globally and causes chronic pain, reducing their quality of life [1, 2]. The pathogenesis of TMJOA comprises the deterioration of articular cartilage, the emergence of subchondral bone osteophytes and lesions, synovial hyperplasia, and angiogenesis [3]. Although the precise pathogenic mechanism of TMJOA remains unknown, current research has demonstrated that an imbalance in the intra-articular antioxidant system is critical in chondrocyte dysfunction and death [4, 5]. Chondrocytes death arises through various mechanisms, such as apoptosis, autophagy, and ferroptosis [6–8]. Chondrocytes ferroptosis has recently been identified as an osteoarthritis (OA) advancement [9], with similarity in abnormal iron metabolism [10, 11], mitochondrial dysfunction [12, 13] and lipid peroxidation [14, 15].

In 2012, Dixon proposed ferroptosis as a new type of programmed cell death [16]. The small molecule erastin impedes cystine intake, leading to L-Glutathione (GSH) depletion and glutathione peroxidase 4 (GPX-4) inactivation, accumulating Fe^2+^, lipid peroxidation, and cell death. Various *in vivo* and *in vitro* investigations have revealed that ferroptosis is vital in several chronic diseases (including traumatic brain injury, intracerebral hemorrhage, carcinogenesis, ischemia-reperfusion injury, and osteoarthritis) and might be a promising therapeutic target [17].

The current therapy for OA primarily involves non-steroidal anti-inflammatory drugs (NSAIDs) [18], which can cause different side effects, such as gastric ulcers [19]. Thus, novel therapeutic alternatives with fewer side effects are urgently required. Plumbagin (PLB), a natural plant extract with anti-inflammatory, antioxidant, and anti-tumor properties [20–22], has been demonstrated to impede the progression of human OA [23]. PLB has been found to preserve cartilage homeostasis and diminish arthritis advancement by decreasing the production of proinflammatory cytokines [24]. However, the underlying mechanism of PLB’s therapeutic effects on TMJOA remains unclear.

This study aimed to investigate the impact of ferroptosis on TMJOA and the potential of PLB as a therapeutic agent for TMJOA and expound its action mechanism. Our hypothesis is that PLB might protect chondrocytes from ferroptosis and relieve TMJOA by regulating oxidative stress. We employed various *in vitro* and *in vivo* models to test the impact of ferroptosis on TMJOA. And PLB displayed significant protective effects against chondrocytes ferroptosis and reduced TMJOA progression by improving antioxidant capacity. RNA-seq and bioinformatics approaches were investigated to examine possible functional signaling pathways to explore mechanism of PLB in regulating ferroptosis, and western blot was used for verification. Our findings implied that PLB could be a promising therapeutic agent for TMJOA and shed light on the pathogenesis of TMJOA from the perspective of ferroptosis and the antioxidant system.

## MATERIALS AND METHODS

### Isolation, culture and identify of rat chondrocytes

Primary chondrocytes were isolated from TMJ and knee joint cartilage of 3-day-old Sprague Dawley (SD) rats as described previously [25]. Briefly, after dissection into pieces, cartilage tissue was digested by 0.25% trypsin containing EDTA (Solarbio, China) for 0.5 h and 0.2% collagenase type II (Beyotime, China) for 4 h. The primary chondrocytes were filtered and resuspended in DMEM/F12 media (Solarbio) with 20% fetal bovine serum (Cyagen, China) and 1% penicillin-streptomycin in a 37° C 5% CO_2_ environment.

Primary chondrocytes were identified using type II collagen (Col2α1) and aggrecan (ACAN) immunofluorescence, as directed by the manufacturer. We only used second-passage chondrocytes to ensure phenotypic integrity.

Recombinant H_2_O_2_ (H1009, Sigma, USA), Ferrostatin-1 (Fer-1) (HY-100579, MCE, USA), and PLB (HY-N1497, MCE) and Mulberroside A (Mul-A) (HY-N0619, MCE) were treated with primary chondrocytes.

### CCK-8 cell viability assay

Primary chondrocytes were seeded at a density of 8000 cells per well in 96-well plates with 100 μL supplemented with 10 μL CCK-8 reagent (Beyotime) and incubated at 37° C for 2 h after the specified treatment. A microplate reader was used to measure absorbance values at 450 nm to determine cell viability (Thermo Fisher Scientific, USA).

### ROS, lipid peroxidation, Fe^2+^assay

DCFH-DA (D6470, Solarbio) was employed to test the levels of reactive oxygen species (ROS) and was assessed using fluorescence microscopy (Ti-S-Fi1C/Nikon, Japan). To detect lipid peroxidation (Liperfluo/Dojindo, Japan) and Fe^2+^ (FerroOrange/Dojindo), chondrocytes were seeded and treated with H_2_O_2_ (500 μM) Fer-1 (2 μM) or PLB (0.5, 1, 2 μM) in 24-well-plates, washed with PBS twice, assayed by laser confocal microscope (LSM 900 with Airyscan 2/Zeiss, Germany).

### GSH and MDA assay

MDA and GSH levels in chondrocytes were measured using the GSH assay kit (Nanjing Jiancheng, China) and the MDA assay kit (Nanjing Jiancheng). Chondrocytes were collected in preparation for ultrasonic lysis. The GSH and MDA contents of protein lysates were then assessed and standardized based on protein concentrations. The BCA protein assay kit is used to determine the concentration of protein (Servicebio, China). By enzyme cycling, GSH reacted with 5,5' -dithio-di-2-nitrobenzoic acid (DTNB) to produce GSSH and stable products. At OD 412 nm, the product can be determined. Different concentrations of standard substances were used to create the standard curve. MDA was detected by reacting it with thiobarbituric acid (TBA) to form MDA-TBA adduct, and the absorbance at 532 nm was measured using a microplate reader.

### JC-1 staining

The mitochondrial membrane potential staining kit (JC-1 kit, Servicebio, China) was used to assess the effects of various treatments on chondrocytes mitochondrial membrane potential. Chondrocytes were seeded into 6-well plates and incubated with JC-1 working solution for 20 min at 37° C in the dark after various treatments. After removing the working solution, it was washed 3 times with PBS to improve detection of fluorescence intensity. Finally, laser confocal microscope (LSM 900 with Airyscan 2/Zeiss) images of red (JC-1 aggregates) and green (JC-1 monomers) fluorescence were acquired.

### Transmission electron microscopy

Cartilage tissue and chondrocytes were fixed for 24 h in 2.5% glutaraldehyde (Solarbio, China) at 4° C. The fixative should then be cleaned with PBS. After 3 times of PBS rinses, the fixed cartilage tissue and chondrocytes were post-fixed for 2 h at room temperature with 2% osmium tetroxide. Cells were washed 3 times with PBS before being dehydrated in a graded series of alcohol (50%, 70%, 80%, 90%, 95%, and 100%) before being embedded in Epon 816 (Electron Microscopy Sciences, USA), and ultrathin sections (60-80 nm) were cut using a Leica ultramicrotome (Leica Microsystems, USA). The prepared sections were then stained twice with uranyl acetate and lead citrate. A transmission electron microscope was eventually used to capture ultrastructural images of the chondrocytes (TEM, HT7800, Hitachi, Japan).

### Quantitative real-time PCR analysis

According to the manufacturer's instructions, the Cell Total RNA Isolation Kit (EZB/EZB-TZ1) was extracted from cultured chondrocytes. The concentration of RNA was determined using a NanoDrop 2000 (Thermo Fisher Scientific, USA), and cDNA was generated using the PrimeScript RT reagent kit (G3330, Servicebio). qRT-PCR was carried out in a Bio-Rad Real-Time System using SYBR Green (GM2007, Servicebio). The 2^−ΔΔCt^ method was used to normalize relative gene expression by β-actin. Supplementary Table 1 contains a list of the primers.

### Western blot analysis

Chondrocytes were treated differently, as previously described. RIPA (Solarbio Biotech, China) and PMSF (NIC Biotech, China) were then used to extract the protein from chondrocytes. For 2 min, an ultrasound was used to lyse the cell lysate—centrifuge for 15 min at 4° C in a high-speed centrifuge. The supernatant was then collected to determine the BCA (Solarbio Biotech, China) protein concentration. It was then diluted with a protein loading buffer (NIC Biotech, China) and heated for 10 min in a 100° C metal bath. SDS-PAGE was used to separate equal amounts of the extracted proteins, which were then transferred to polyvinylidene fluoride (PVDF, NIC Biotech, China) membranes. The membranes were blocked for 1 h at room temperature with 5% skim milk before incubating with specific primary antibodies overnight at 4° C. The membranes were then incubated for 1 h at room temperature with corresponding HRP-conjugated secondary antibodies (AST, USA). The protein bands communicated with the ECL solvent (Servicebio, China) before being visualized by the ChemiDocTM XRS System (Bio-Rad, USA). Using ImageJ software, protein expression levels were measured and quantified using β-actin as an internal control. Primary antibodies include Col2α1, ACAN, MMP-13, Adamts-5, GPX-4, SLC7A11, ACSL4, COX-2, ERK, p-ERK, P38, p-P38, JNK, p-JNK (Cell Signaling Technology, USA).

### RNA-seq and bioinformatics analysis

The same method was used to extract and purify total RNA from cell samples according to the manufacturer's instructions (Invitrogen, USA). Total RNA was extracted from H_2_O_2_-induced chondrocytes in the CON group, H_2_O_2_ group treated with H_2_O_2_, and PLB group treated with 2 μM PLB and sequenced by Guangdong Magigene, Co., Ltd. The fold change > 1.5 and P value 0.05 thresholds were used to identify differentially expressed genes (DEGs). DEG enrichment analysis was also carried out using Gene Ontology (GO) and Kyoto Encyclopedia of Genes and Genomes (KEGG) pathways enrichment analysis. The use of KEGG database resources, as well as molecular-level information and expressed gene and fold change data obtained from biosystems analysis, was used to understand high-level function. The differentially expressed genes at different levels of the KEGG pathways were counted to identify metabolic and signaling pathways. Understanding high-level functions was done by utilizing the KEGG database resource, as well as molecular-level information and expressed genes with fold change data obtained from biological system analysis. The differentially expressed genes at different levels of the KEGG pathways were counted to identify metabolic and signaling pathways.

### Animal experiment

The First Affiliated Hospital of Nanchang University provided 6-week-old male Sprague Dawley rats. An experimental TMJOA model was established in the rats by performing unilateral anterior crossbite (UAC) surgery after anesthesia with an intraperitoneal injection of pentobarbital (30 mg/kg) [26]. 24 rats were randomly divided into 3 groups (n = 8): the CON, UAC, and Fer-1 (1 mg/kg). Additionally, 40 rats were divided into 5 groups: the CON group, the UAC group, the PLB0.5 group, the PLB1 group, and the PLB2 group. The PLB0.5, PLB1, and PLB2 groups received intra-articular injections of 0.5 mg/kg, 1 mg/kg, and 2 mg/kg PLB, respectively. The CON and UAC groups of rats received the same volume of physiological saline intra-articularly. In week 4 of intra-articularly, the rats were injected into the joint cavity every 3 days. All rats were euthanized at the end of 8 weeks, and TMJ tissue was collected for further research.

### Micro-CT

A micro-CT system (SkyScan 1176; Bruker Corp., Germany) was used to scan the joints at 385 A, 65 kV, and a thickness of 9 m per slice. CTAn was used to analyze radiographs for relative parameters such as bone volume fraction (BV/TV), trabecular thickness (Tb.Th), trabecular separation (Tb.Sp), and trabecular number (Tb.N). CTvox software was used to generate 3D images for morphological analysis.

### Histology and immunohistochemical assay

The TMJ joint tissue underwent paraformaldehyde immersion for 24 h, followed by decalcification with 10% EDTA for 4 weeks. Afterward, the tissue was embedded in paraffin and cut into 4 mm thick slices, stained with H&E and Safranin O/fast green. The Osteoarthritis Research Society International (OARSI) score system was used to assess the osteoarthritis score of the joint tissue. The tissue sections were inactivated for peroxidase using 0.3% H_2_O_2_ and sealed with 3% BSA for antigen before being incubated overnight in a 4° C refrigerator with pre-configured primary antibodies. On the second day, the sections were incubated for 1 h at room temperature with fluorescein-anti-rabbit secondary antibody before being photographed and recorded with a fluorescence microscope.

To prepare the TMJ joint tissues, they were fixed in 4% PFA for 24 h and decalcified with 10% EDTA (PH = 7.4) for 4 weeks. The tissues were then embedded in paraffin and sectioned in the sagittal plane to a thickness of 4 μm. Subsequently, the sections were stained with hematoxylin, eosin (H&E), and Safranin O/fast green. The Osteoarthritis Research Society International (OARSI) score system was used blindly to evaluate the severity of cartilage degeneration in the rat TMJOA model. After deparaffinization and rehydration, the sections were treated with 0.3% H_2_O_2_ for 10 min, followed by 0.3% Triton X-100 for 30 min and blocked with 5% BSA for 1 h. Primary antibodies against GPX-4 were then added and incubated overnight at 4° C, followed by incubation with the goat anti-rabbit secondary antibody. Finally, the samples were stained with hematoxylin and visualized using a fluorescence microscope (Olympus, Japan).

### Statistical analysis

The experimental results were expressed as mean ± standard deviation (SD). Statistical analysis was conducted using SPSS 20.0. Each experiment was performed in triplicate. The statistical significance of differences among multiple groups was determined using a one-way analysis of variance (ANOVA) followed by Tukey's test. A P-value of less than 0.05 was considered to be statistically significant.

## RESULTS

### Fer-1 alleviated TMJOA progression in a rat UAC model

**Figure 1:**
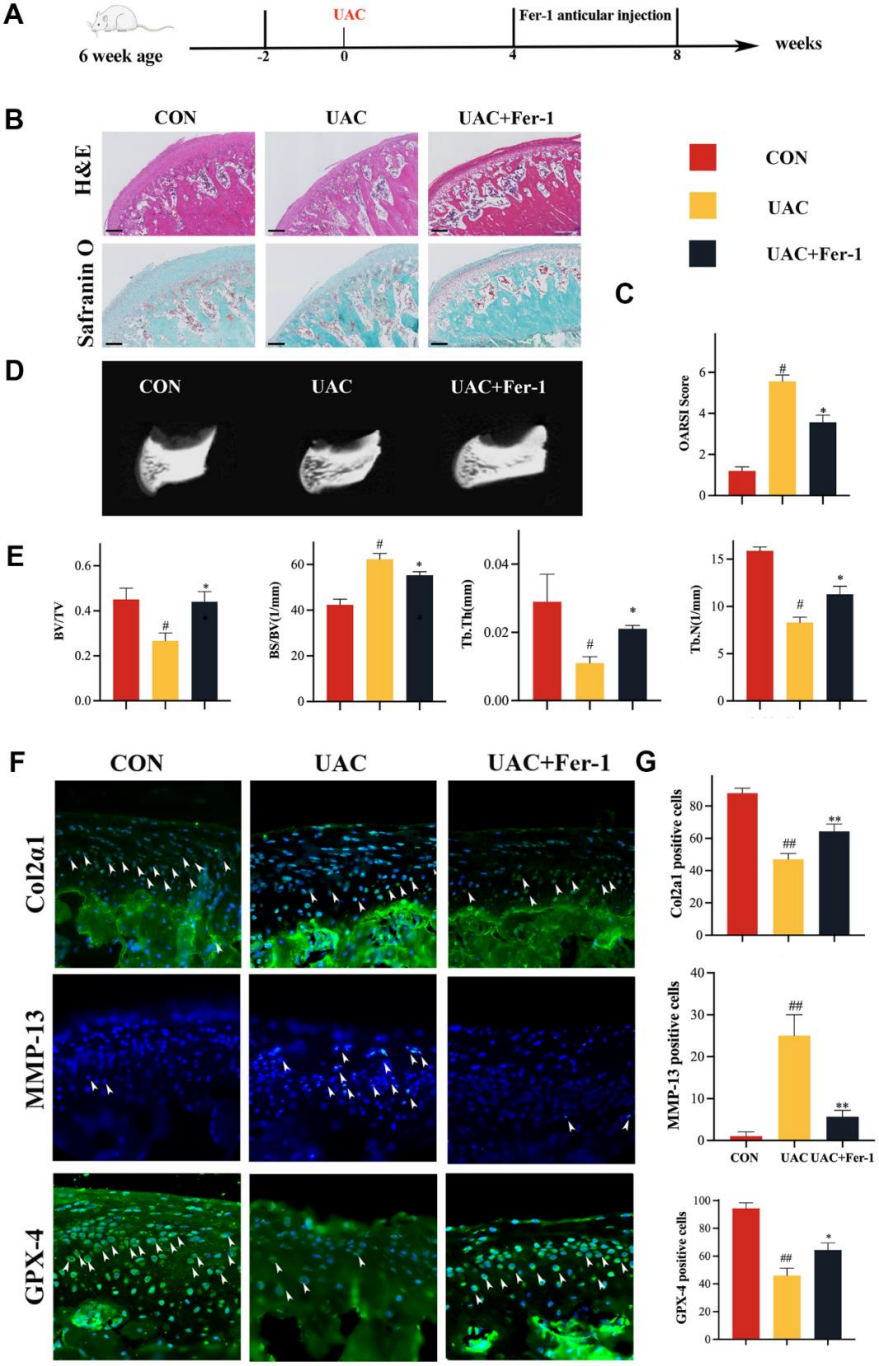
**Fer-1 alleviated TMJOA progression in a rat UAC model.** (**A**) Schematic model of the time course for establishment of the unilateral anterior crossbite (UAC) model of TMJOA rat treated with Fer-1 (1 mg/kg) by articular injection. (**B**) Representative H&E and Safranin O/fast green staining of control and UAC-induced TMJOA rat treated with/without Fer-1. Scale bars, 200 μm. (**C**) Osteoarthritis Research Society International (OARSI) score evaluated based on Safranin O/fast green staining. n = 3. (**D**) Representative micro-CT of control and UAC-induced TMJOA rat treated with/without Fer-1. (**E**) Quantitative analysis of subchondral bone parameters of control and UAC-induced TMJOA rat treated with/without Fer-1. (**F**) Representative immunofluorescence staining of Col2α1, MMP-13 and GPX-4 of control and UAC-induced TMJOA rat treated with/without Fer-1. Arrow heads indicated positive cells. n = 3. Scale bars, 50 μm. (**G**) Quantitative analysis of immunofluorescence staining of Col2α1, MMP-13 and GPX-4 of control and UAC-induced TMJOA rat treated with/without Fer-1. (All quantified data are shown as mean ± SEM; #p < 0.05, ##p < 0.01, *p < 0.05, **p < 0.01, by one-way ANOVA followed by the Tukey-Kramer test, #: CON vs UAC, *: UAC vs UAC+Fer-1).

In order to explore the role of ferroptosis in the pathogenesis of TMJOA, fer-1 is used to interfere with the TMJOA model, we conducted a study for investigating cartilage changes and ferroptosis-related indices in three groups: CON, UAC, and UAC+Fer-1. Fer-1 (1mg/kg) was administered injected into the TMJ joint cavity when 4 weeks after UAC surgery. At 8 weeks after surgery, cartilage specimens were collected (Figure 1A). H&E staining showed a smooth surface of normal cartilage and overall cartilage thinning after UAC surgery, while staining with Safranin O demonstrated proteoglycan loss following UAC surgery (Figure 1B). Nevertheless, articular cartilage degeneration was dramatically reduced in the Fer-1 group, and the OARSI score was significantly lower when compared to the UAC group (Figure 1C). Additionally, Micro-CT imaging revealed that Fer-1 significantly inhibited the subchondral bone resorption induced by UAC (Figure 1D, 1E).

We then assessed the levels of Col2α1 and matrix metalloproteinase-13 (MMP-13) in the cartilage specimens using immunofluorescence staining. Our results demonstrated significantly higher levels of Col2α1 and significantly lower levels of MMP-13 in the Fer-1 group compared to the UAC group (Figure 1G, 1H). We also investigated the effect of Fer-1 on the activity of GPX-4 and the morphology of mitochondria. As shown in Figure 1F, mitochondrial morphological UAC-induced TMJOA rat, mitochondria become smaller and the mitochondrial membrane is ruptured. Our results demonstrated that GPX-4 activity was enhanced after Fer-1 intervention (Figure 1G, 1H), and Fer-1 improved the morphology of UAC-treated mitochondria, resulting in reduced bilayer density (Supplementary Figure 2). These results suggest that Fer-1 alleviates the progression of TMJOA primarily by inhibiting articular cartilage breakdown and GPX-4 inactivation.

### Fer-1 suppressed H_2_O_2_-induced catabolism and ferroptosis in chondrocytes

**Figure 2:**
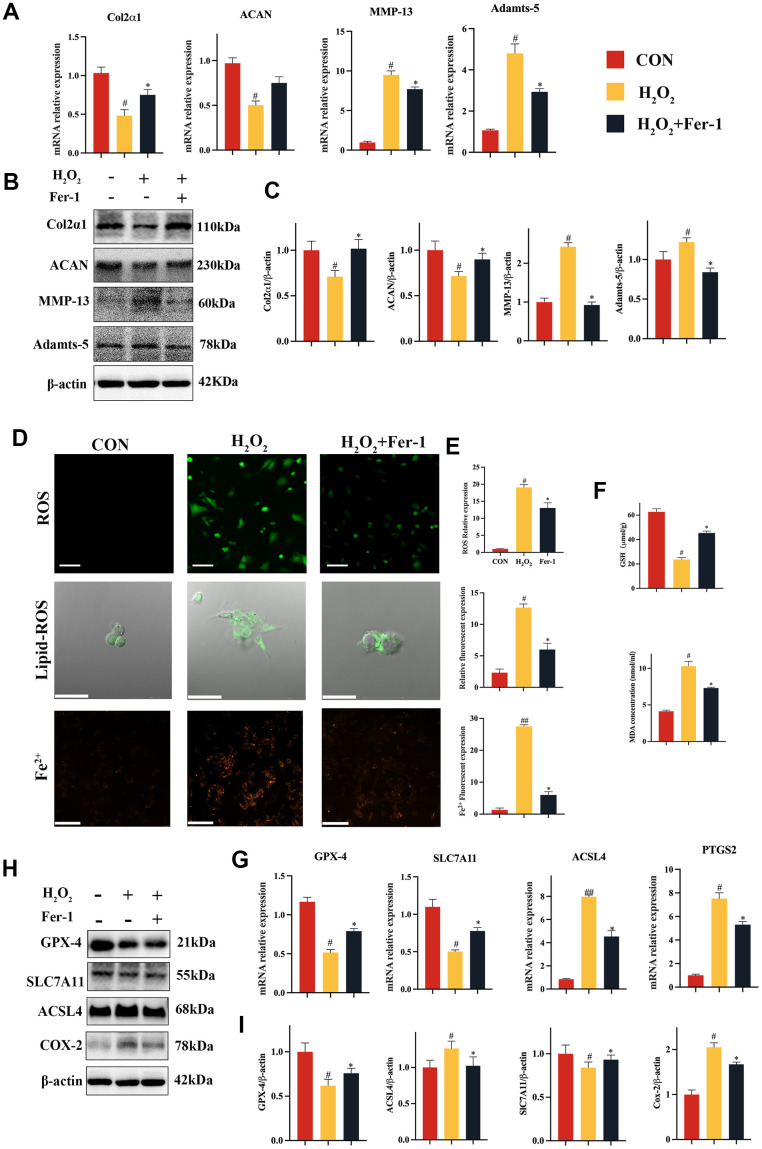
**Fer-1 suppressed H_2_O_2_-induced catabolism and ferroptosis of chondrocytes.** (**A**) Quantitative qRT-PCR of the gene expression of Col2α1, ACAN, MMP-13 and Adamts-5 under Fer-1 (2 μM) and H_2_O_2_ (500 μM) cultured conditions treatment. (**B**) Western blot analyses of Col2α1, ACAN, MMP-13 and Adamts-5 under Fer-1 and H_2_O_2_ cultured conditions treatment. (**C**) Quantitative western blot of Col2α1, ACAN, MMP-13 and Adamts-5 under Fer-1 and H_2_O_2_ cultured conditions treatment. n = 3. (**D**) Representative ROS, Lipid-ROS and Fe^2+^ fluorescence detection under Fer-1 and H_2_O_2_ cultured conditions treatment. (**E**) Quantitative ROS, Lipid-ROS and Fe^2+^ fluorescence under Fer-1 and H_2_O_2_ cultured conditions treatment. n = 3. ROS and Fe^2+^: Scale bars, 50 μm. Lipid-ROS: Scale bars, 100 μm. (**F**) Quantitative analysis of GSH and MDA under Fer-1 and H_2_O_2_ cultured conditions treatment. (**G**) Quantitative qRT-PCR of the gene expression of GPX-4, SLC7A11, ACSL4 and COX-2 under Fer-1 and H_2_O_2_ cultured conditions treatment. (**H**) Western blot analyses of GPX-4, SLC7A11, ACSL4 and COX-2 under Fer-1 and H_2_O_2_ cultured conditions treatment. (**I**) Quantitative western blot of GPX-4, SLC7A11, ACSL4 and COX-2 under Fer-1 and H_2_O_2_ cultured conditions treatment. n = 3. (All quantified data are shown as mean ± SEM; #p < 0.05, ##p < 0.01, *p < 0.05, **p < 0.01, by one-way ANOVA followed by the Tukey-Kramer test, #: CON vs H_2_O_2_, *: H_2_O_2_ vs Fer-1+ H_2_O_2_).

In this study, we aimed to investigate the role of ferroptosis in the progression TMJOA. To this end, we conducted experiments *in vitro* to validate the effect of the ferroptosis inhibitor Fer-1 on chondrocytes catabolism. To evaluate the effect of Fer-1 (2 μM) on H_2_O_2_-induced chondrocytes catabolism *in vitro*, we isolated primary chondrocytes from infant rats (Supplementary Figure 1A) and cultured them in media supplemented with or without Fer-1 in the presence of H_2_O_2_. Our results showed that Fer-1 inhibited the reduction in Col2a1 and ACAN and the elevation of MMP-13 and Adamts-5 gene mRNA levels caused by H_2_O_2_ (Figure 2A). Western blot analysis (Figure 2B, 2C) confirmed these findings, indicating that Fer-1 regulates cartilage homeostasis mainly by inhibiting H_2_O_2_-induced catabolism in chondrocytes.

After that, we looked into the effect of Fer-1 on H_2_O_2_-induced chondrocytes ferroptosis *in vitro*. We examined ferroptosis indicators, including ROS, Lipid-ROS and Fe^2+^ (Figure 2D, 2E). Our results showed that H_2_O_2_ caused an elevation of ROS, Lipid ROS and Fe^2+^ effectively inhibited by Fer-1 (Figure 2D). Lipid peroxidation with accumulation and GSH reduction, which characterizes the process of ferroptosis, was also significantly inhibited by Fer-1, as evidenced by the suppression of MDA (Figure 2F). Furthermore, our data from qRT-PCR and western blot showed that Fer-1 could restore SLC7A11, and GPX-4 activity while suppressed ACSL4 and COX-2 expression (Figure 2G–2I). Lastly, we observed the morphology of mitochondria and found that Fer-1 was able to restore the abnormal morphology caused by H_2_O_2_ (Supplementary Figure 3), which was consistent with the results of our *in vivo* experiments.

Our findings showed that Fer-1 maintained cartilage homeostasis by reducing H_2_O_2_-induced ferroptosis in chondrocytes. These findings provided valuable insights into the potential use of Fer-1 as a therapeutic strategy for treating TMJOA and other related diseases.

### PLB alleviated TMJOA progression in a rat UAC model

**Figure 3:**
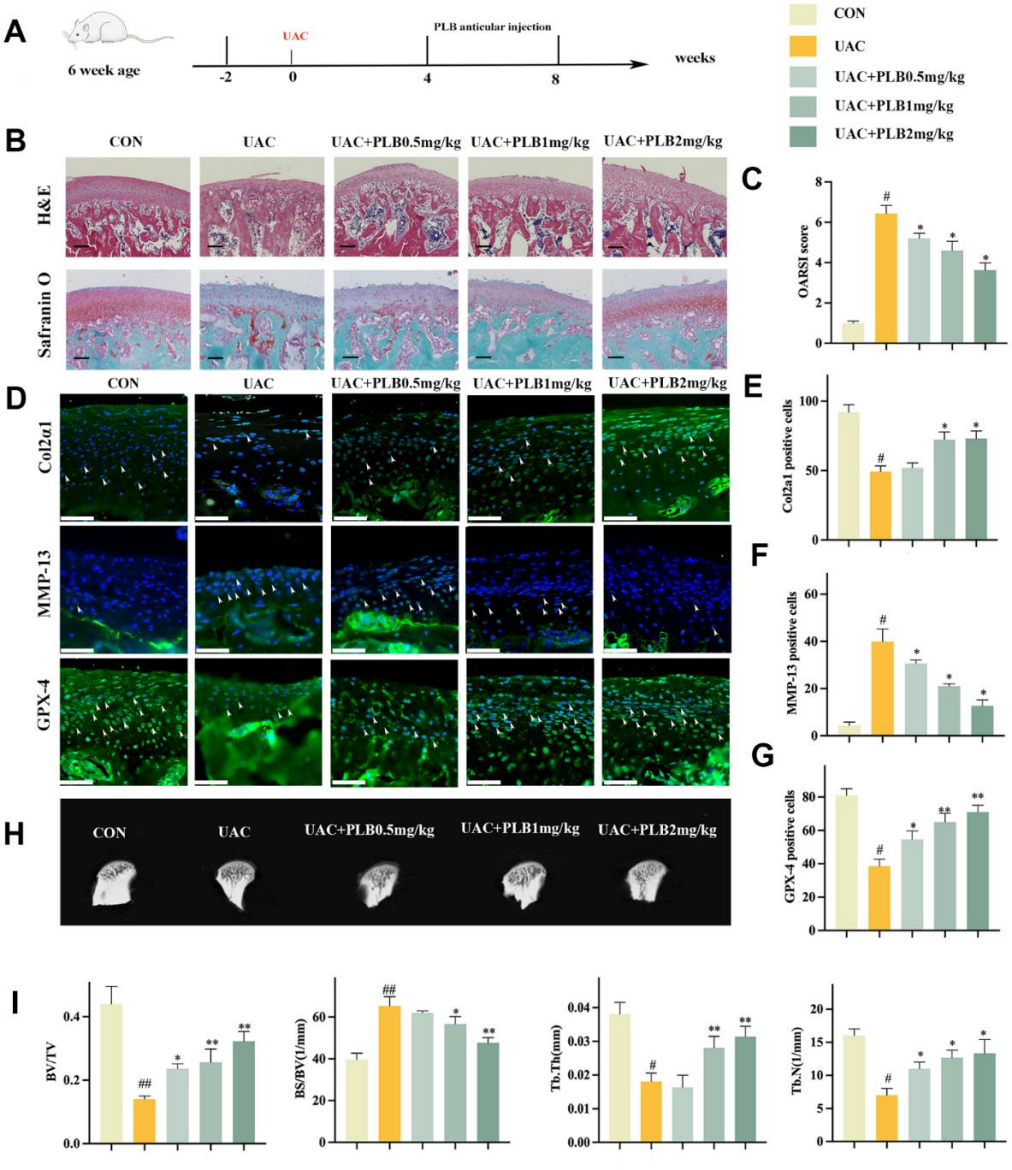
**PLB alleviated TMJOA progression and cartilage degeneration in the rat UAC model.** (**A**) Schematic model of the time course for establishment of UAC model of TMJOA rat treated with PLB by articular injection. (**B**) Representative H&E and Safranin O/fast green staining of control and UAC-induced TMJOA rat treated with/without different PLB (0.5, 1, 2 mg/kg). Scale bars, 200 μm. (**C**) Osteoarthritis Research Society International (OARSI) score evaluated based on Safranin O/fast green staining. n = 3. (**D**) Representative immunofluorescence staining of Col2α1, MMP-13 and GPX-4. Arrow heads indicated positive cells. n = 3. Scale bars, 50 μm. (**E**–**G**) Quantitative analysis of immunofluorescence staining of Col2α1, MMP-13 and GPX-4. (**H**) Representative Micro-CT of control and UAC-induced TMJOA rat treated with/without PLB. (**I**) Quantitative analysis of subchondral bone parameters of control and UAC-induced TMJOA rat treated with/without PLB. (All quantified data are shown as mean ± SEM; #p < 0.05, ##p < 0.01, *p < 0.05, **p < 0.01, by one-way ANOVA followed by the Tukey-Kramer test, #: CON vs UAC, *: UAC vs UAC+PLB).

To investigate the potential role of PLB in the progression of TMJOA, we conducted a series of experiments examining the application of PLB at varying concentrations (i.e., CON, UAC, UAC+PLB 0.5 mg/kg, UAC+PLB 1 mg/kg, and UAC+PLB 2 mg/kg) and its effect on cartilage changes and indicators related to ferroptosis. Specifically, PLB was injected into the TMJ joint cavity starting from 4 weeks after UAC surgery and cartilage samples were taken 8 weeks after surgery (Figure 3A).

H&E staining revealed the presence of a smooth surface in normal cartilage, while overall cartilage thinning was observed after UAC. Furthermore, staining with Safranin O indicated proteoglycan loss after UAC (Figure 3B). Safranin O was assessed using OARSI score, a well-acknowledged scoring system for evaluating the severity of OA. Micro-CT showed that PLB significantly inhibited subchondral bone resorption caused by UAC (Figure 3H, 3I). Immunofluorescence staining revealed that the PLB group had significantly higher Col2α1 and much lower levels of the reduction MMP-13 than the UAC group (Figure 3D–3G). Taken together, these results suggested that PLB supplementation could slow the course of TMJOA by preventing articular cartilage catabolism.

### PLB suppressed H_2_O_2_-induced catabolism and ferroptosis in chondrocytes

**Figure 4:**
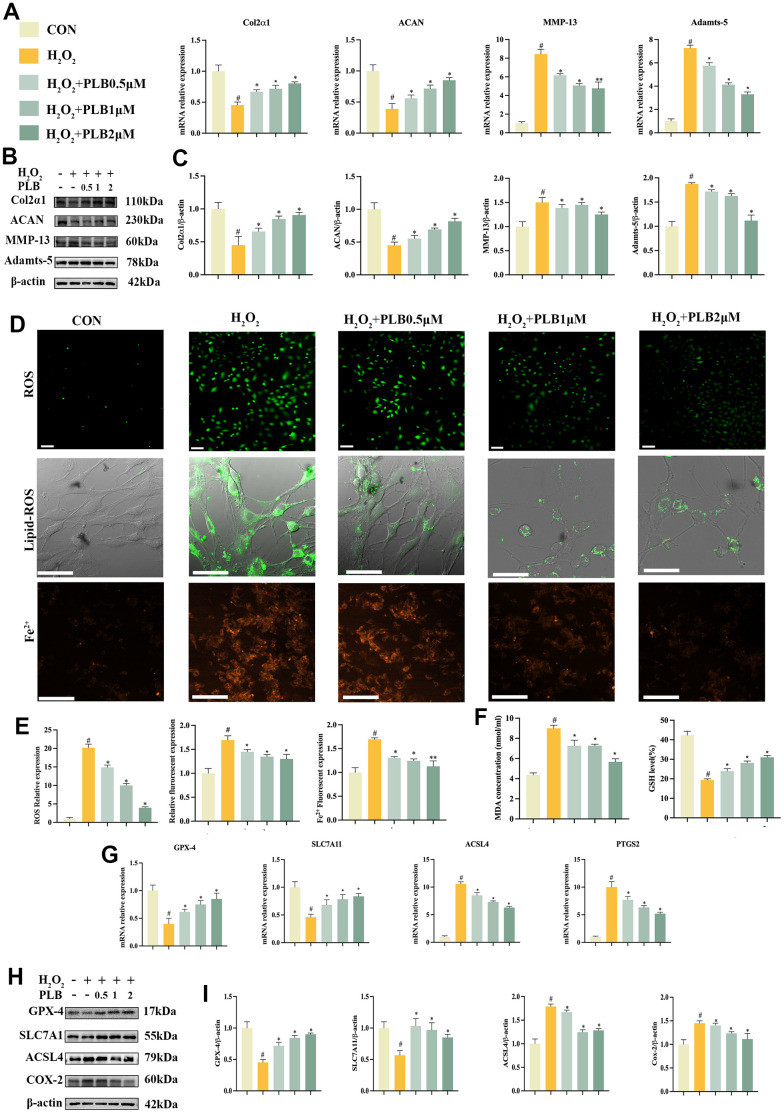
**PLB suppressed H_2_O_2_-induced catabolism and ferroptosis of chondrocytes.** (**A**) Quantitative qRT-PCR of the gene expression Col2α1, ACAN, MMP-13 and Adamts-5 under PLB (0.5, 1, 2 μM) and H_2_O_2_ (500 μM) cultured conditions treatment. (**B**) Western blot analyses of Col2α1, ACAN, MMP-13 and Adamts-5 under PLB and H_2_O_2_ cultured conditions treatment. (**C**) Quantitative western blot of Col2α1, ACAN, MMP-13 and Adamts-5 under PLB and H_2_O_2_ cultured conditions treatment. n = 3. (**D**) Representative ROS, Lipid-ROS and Fe^2+^ fluorescence detection under PLB and H_2_O_2_ cultured conditions treatment. (**E**) Quantitative analysis of ROS, Fe^2+^, Lipid-ROS under PLB and H_2_O_2_ cultured conditions treatment. ROS and Fe^2+^: Scale bars, 50 μm. Lipid-ROS: Scale bars, 100 μm. (**F**) Quantitative analysis of MDA and GSH under PLB and H_2_O_2_ cultured conditions treatment. (**G**) Quantitative qRT-PCR of the gene expression and (**H**) western blot analyses of GPX-4, SLC7A11, ACSL4 and COX-2 under PLB and H_2_O_2_ cultured conditions treatment. (**I**) Quantitative western blot of GPX-4, SLC7A11, ACSL4 and COX-2 under PLB and H_2_O_2_ cultured conditions treatment. n = 3. (All quantified data are shown as mean ± SEM; #p < 0.05, ##p < 0.01, *p < 0.05, **p < 0.01, by one-way ANOVA followed by the Tukey-Kramer test, #: CON vs H_2_O_2_, *: H_2_O_2_ vs PLB+ H_2_O_2_).

To gain further insight into the role of PLB in cartilage homeostasis and chondrocytes ferroptosis, we conducted experiments *in vitro* to validate our findings. Initially, we investigated the impact of PLB on H_2_O_2_-triggered chondrocyte catabolism *in vitro*. Chondrocytes were cultured in PLB (0.5 μM, 1 μM, 2 μM) medium supplemented with different concentrations of H_2_O_2_, and we analyzed genes and proteins associated with chondrogenic metabolism. Our results indicated that PLB effectively obstructed the decrease of Col2α1 and ACAN while inhibited the rise of MMP-13 and Adamts-5 gene mRNA levels caused by H_2_O_2_ (Figure 4A). Additionally, western blot analysis (Figure 4B, 4C) supported the results of qRT-PCR. In conclusion, these findings suggested that PLB regulated cartilage homeostasis mainly by impeding H_2_O_2_-induced chondrocyte catabolism.

Furthermore, we investigated the effect of PLB on H_2_O_2_-induced chondrocytes ferroptosis *in vitro*. We used the same treatment protocol as before and assessed indicators related to ferroptosis. Firstly, ferroptosis often involves the Fenton reaction, which intracellularly generates ROS, Lipid-ROS and Fe^2+^. Immunofluorescence analyses demonstrated that H_2_O_2_ exposure caused an increase in ROS, Lipid-ROS and Fe^2+^, which PLB effectively suppressed (Figure 4D, 4E). Moreover, lipid peroxidation occurs in the process of ferroptosis, producing lipid peroxidation products such as MDA. Our results demonstrate that PLB significantly obstructed the accumulation of these products.

Additionally, our data from qRT-PCR and western blot showed that Fer-1 could restore the activity of GSH, SLC7A11, and GPX-4 while suppressing ACSL4 and COX-2 expression (Figure 4G–4I). Finally, we also observed changes in mitochondrial membrane potential, which PLB restored (Supplementary Figure 4). In conclusion, these results prove that PLB mainly regulates cartilage homeostasis by impeding H2O2-induced iron-dependent cell death (ferroptosis) in chondrocytes.

### PLB suppressed H_2_O_2_-induced catabolism and ferroptosis in chondrocytes via inhibiting MAPK pathways

**Figure 5:**
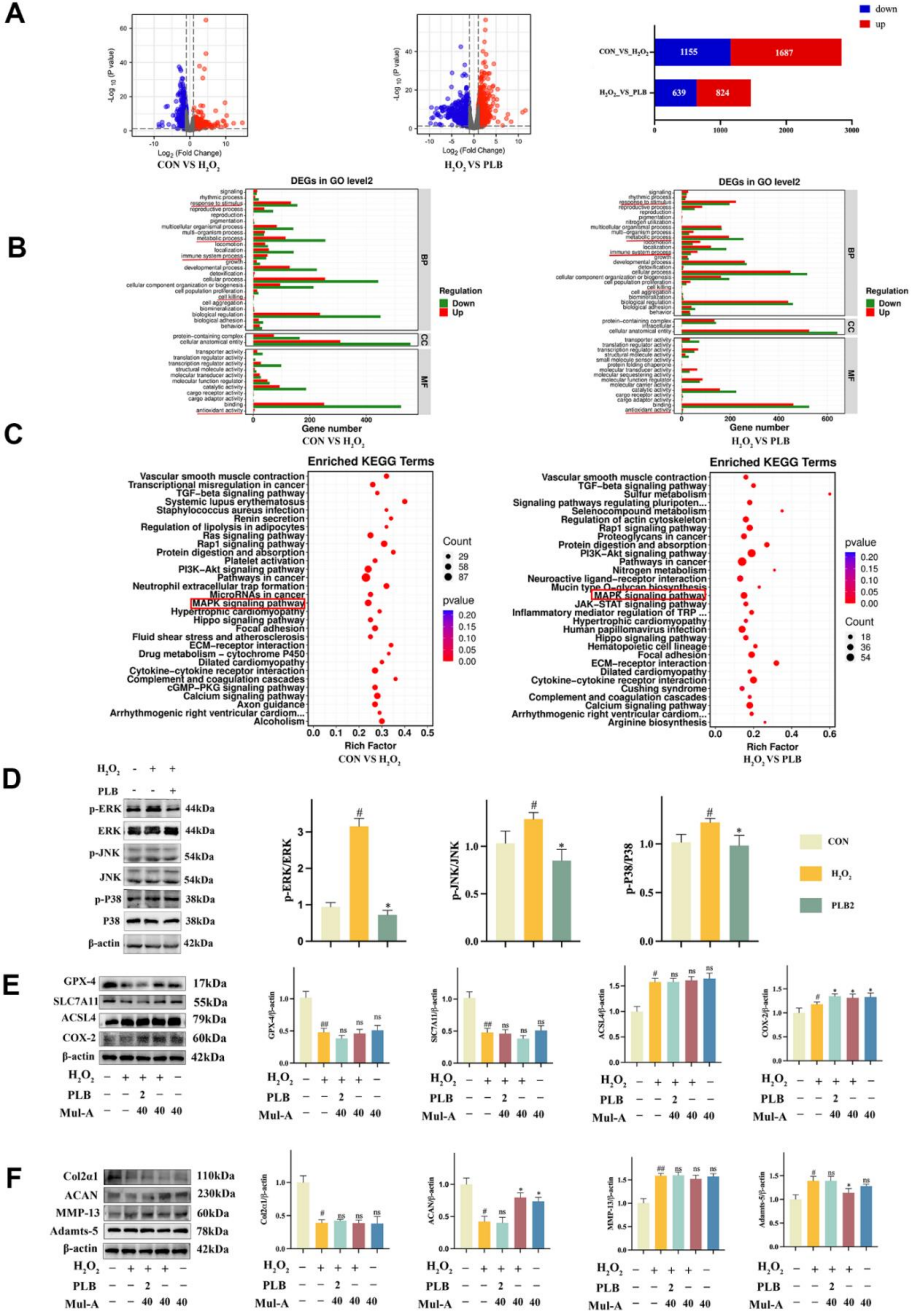
**PLB regulated ferroptosis by inhibiting chondrocytes catabolism via MAPK pathways.** (**A**) The changes of mRNA expression were shown on volcano charts and bar chart. (**B**) The GO analysis. (**C**) The KEGG analysis. (**D**) Western blot analyses of ERK, p-ERK, JNK, p-JNK, P38 and p-P38 under PLB (2 μM) and H_2_O_2_ (500 μM) cultured conditions treatment and quantification. n = 3. (**E**) Western blot analyses of GPX-4, SLC7A11, ACSL4 and COX-2 under PLB, H_2_O_2_ and Mul-A cultured conditions treatment. (**F**) Quantitative western blot of Col2α1, ACAN, MMP-13 and Adamts-5 under PLB and H_2_O_2_ cultured conditions treatment. n = 3 (All quantified data are shown as mean ± SEM; #p < 0.05, ##p < 0.01, *p < 0.05, **p < 0.01, by one-way ANOVA followed by the Tukey-Kramer test, #: CON vs H_2_O_2_, *: H_2_O_2_ vs PLB+ H_2_O_2_).

To further investigate the influence of PLB (2 μM) on cartilage metabolism and ferroptosis, we conducted RNA-seq and bioinformatics analyses to identify target pathways, which were subsequently validated using western blot [27]. Initially, RNA was extracted from CON, H_2_O_2_, and PLB samples, and pairwise comparisons were performed to identify differentially expressed genes. We identified up-regulated and down-regulated genes for CON compared to H_2_O_2_, CON to PLB, and PLB to H_2_O_2_ (Figure 5A).

We then conducted GO and KEGG analyses on these differentially expressed genes. The results of GO analysis indicated that PLB intervention in chondrocytes was associated with cell killing and oxidative stress (Figure 5B). KEGG analysis revealed 30 pathways associated with PLB regulation, with the MAPK signaling pathways ultimately selected for further investigation based on relevant literature (Figure 5C).

The results of western blot analysis confirmed that the H_2_O_2_ signaling pathways were able to activate the MAPK signaling pathways. That intervention with PLB inhibited the activation of MAPK (Figure 5D). To further validate the role of PLB and the MAPK signaling pathways, we utilized the agonist Mul-A to investigate cartilage characterization. In contrast, using agonists counteracted the therapeutic effect of PLB on H_2_O_2_-stimulated cartilage (Figure 5E, 5F).

In conclusion, these results indicated that the MAPK signaling pathways played a key role in PLB therapy of H_2_O_2_-stimulated cartilage. Our findings shed light on the mechanisms underlying PLB's effect on cartilage metabolism and ferroptosis and may aid in identifying prospective therapeutic targets for treating cartilage-related illnesses.

## DISCUSSION

TMJOA can cause severe pain in the oral and maxillofacial region, with an increased prevalence in older persons [28–30]. Previous studies have shown that TMJOA is a disease associated with oxidative stress [5, 31]. Ferroptosis and oxidative stress are intimately associated [32], and it has been shown that chondrocytes ferroptosis can speed up the development of osteoarthritis [9]. Guo et al. [33] discovered that PLB could prevent H_2_O_2_-induced chondrocyte mortality by decreasing oxidative stress, lipid peroxidation, and inflammation. However, it is unknown whether PLB defends chondrocytes by preventing ferroptosis. Our study first proved that H_2_O_2_ could victim chondrocytes to oxidative stress. Moreover, H_2_O_2_ can also lead to the ferroptosis of chondrocytes by inhibiting GSH, SLC7A11, and GPX-4, promoting overexpression of ACSL4, COX-2, and MDA, and causing ROS, Lipid-ROS, and Fe^2+^ to accumulate. These findings suggest that H_2_O_2_ induces ferroptosis in chondrocytes. Also, the ferroptosis inhibitor Fer-1 prevented OA-like alterations in chondrocytes caused by H_2_O_2_, indicating that inhibiting ferroptosis reduces oxidative stress and the matrix degradation of chondrocytes. Second, Fer-1 could avert the cartilage matrix degradation in UAC-treated animal models by boosting the production of MMP-13 and lowering the creation of Col2α1. It is important to note that Fer-1 could prevent the decrease of GPX-4 in UAC-induced TMJOA. The results were supported by *in vitro* studies, which showed that the progression of TMJOA could be slowed by inhibiting chondrocyte ferroptosis.

According to one study, PLB reduced apoptosis in nucleus pulposus cells by reducing oxidative stress and lipid peroxidation caused by H_2_O_2_ [34]. However, few studies examined the correlation between PLB and ferroptosis. According to our experiments, PLB inhibited chondrocyte ferroptosis and alleviated the progression of TMJOA. PLB boosted GPX-4 and SLC7A11 expression while inhibiting ACSL4, COX-2, and Lipid-ROS expression. It also reduced MDA and Fe^2+^ accumulation, demonstrating that PLB could diminish H_2_O_2_-induced chondrocyte inflammation and matrix destruction via reducing chondrocyte ferroptosis. To confirm the protective effect of PLB on chondrocytes, we created a TMJOA model in rats using UAC. At the same time, others have found that PLB could inhibit the progression of DMM-induced mouse OA models, including inhibiting cartilage matrix degradation, inhibiting subchondral bone absorption and reducing OARSI scores [23]. Our experimental results supported this conclusion, which explained how PLB might protect subchondral bone. Subchondral bone angiogenesis is critical in osteoarthritis [35–37]. PLB decreased tumor angiogenesis in endothelial cells via the Ras signaling pathways mediated by VEGF receptor 2 [38]. PLB might slow the progression of osteoarthritis by inhibiting the formation of subchondral bone vessels, but this needs to be tested further. Finally, when H_2_O_2_ was used to induce ferroptosis, PLB significantly improved the mitochondrial structure and restored mitochondrial membrane potential. The results of our study further confirmed that plumbagin protects mitochondria [39]. In conclusion, our findings show that chondrocyte ferroptosis plays a vital role in the pathogenesis and progression of TMJOA, and that PLB can protect chondrocytes and slow the progression of OA by reducing chondrocyte ferroptosis. MAPK signal pathways regulate multiple physiological processes and responses to oxidative stress [40–42]. MAPK signaling is also involved in ferroptosis initiation. As a result of MAPK inhibition, especially p38 and JNK, AML cells become insensitive to erastin [43]. A study by Poursaitidis et al. [44] consistently showed that inhibiting MAPK signaling prevented ferroptosis in lung cancer cells.

According to western blot examination, PLB lowered the phosphorylation levels of ERK, JNK, and P38 in H_2_O_2_-stimulated chondrocytes. To further verify the relationship between PLB and MAPK signaling pathways, inhibitors and activators of MAPK were used to intervene. The results showed that inhibitors can inhibit the degradation of cartilage matrix proteins by inhibiting ferroptosis, which was similar to the function of PLB. While as a consequence of activating the MAPK pathways, Mul-A activated the chondrocyte therapeutic effect of PLB. The MAPK signaling pathways have been implicated in the progression of TMJOA and is considered a major signaling pathways that regulates the disease [45–47]. The P38MAPK signaling pathways, for example, can stimulate the chemokine CX3CL1, worsening the course of TMJOA [48]. PLB reduced the development of periodontitis in rats by inactivating MAPK and lowering the production of inflammatory factors [49]. In our current study, PLB could downregulate matrix degradation and ferroptosis by inhibiting MAPK activation, thereby reducing joint damage caused by TMJOA.

There should be a few restrictions put forth. First, rather than using a human model, the effect of PLB on TMJOA is based on a rat model. Future research will also enable a better understanding of the optimal dose, length, course, duration of treatment, and long-term effects. Second, unlike apoptosis, ferroptosis detection does not yet have a gold standard approach. However, we have confirmed that H_2_O_2_ induces ferroptosis in chondrocytes in line with the standards of ferroptosis provided by the Cell Death Nomenclature Committee.

## CONCLUSIONS

**Figure 6:**
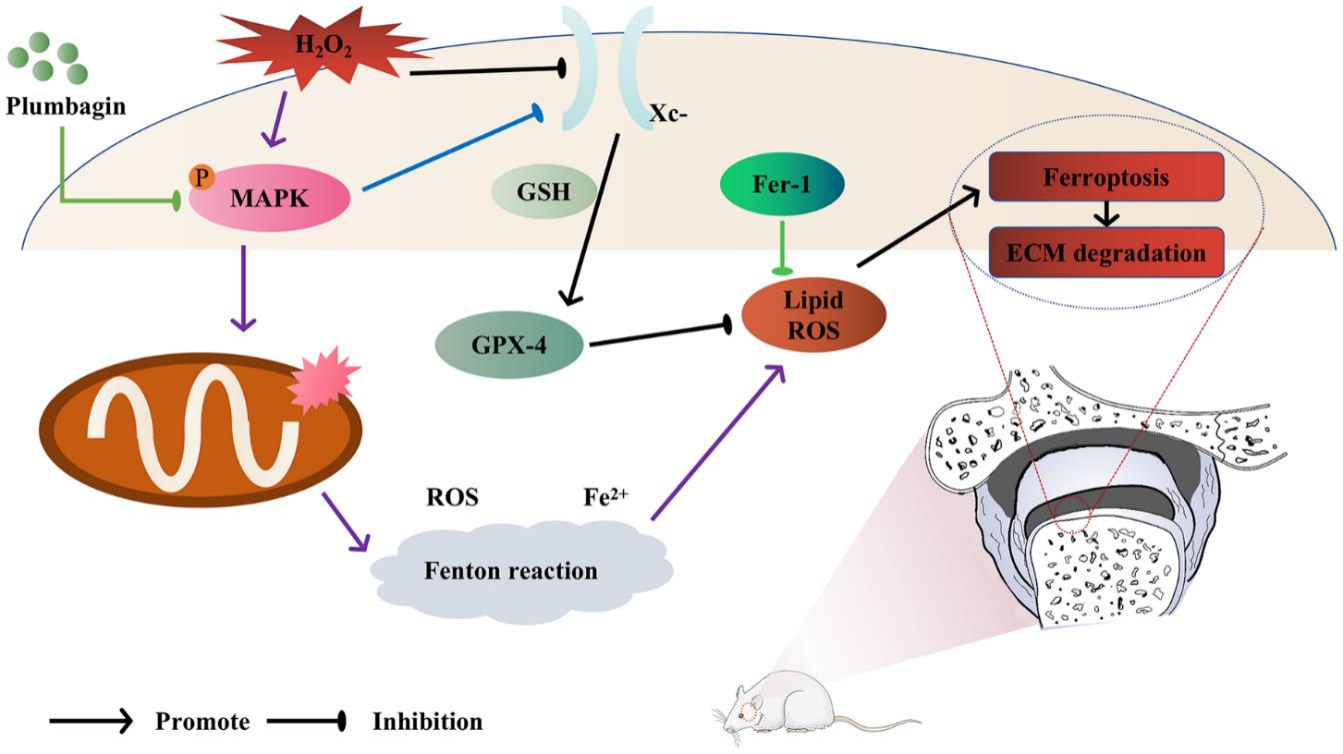
Model of PLB ameliorates TMJOA progression by inhibiting chondrocytes ferroptosis in MAPK pathways.

In conclusion, our research company findings have demonstrated that PLB has a preventive benefit on TMJOA both *in vitro* and *in vivo*. Also, we have shown that PLB slowed down extracellular matrix deterioration by lowering chondrocytes ferroptosis when chondrocytes were stimulated with H_2_O_2_. Furthermore, we demonstrated that PLB exerts its anti-ferroptosis and anti-inflammatory effects via the MAPK pathways (Figure 6). The study's findings are a major step forward in our understanding of this disorder, and they offer promise for developing effective medicines to enhance patient outcomes.

## Supplementary Material

Supplementary Figures

Supplementary Table 1

## References

[1] Serrano-Muñoz D, Beltran-Alacreu H, Martín-Caro Álvarez D, Fernández-Pérez JJ, Aceituno-Gómez J, Arroyo-Fernández R, Avendaño-Coy J. Effectiveness of Different Electrical Stimulation Modalities for Pain and Masticatory Function in Temporomandibular Disorders: A Systematic Review and Meta-Analysis. J Pain. 2023; 24:946–56. 10.1016/j.jpain.2023.01.016 36801166

[2] Brooks SL, Westesson PL, Eriksson L, Hansson LG, Barsotti JB. Prevalence of osseous changes in the temporomandibular joint of asymptomatic persons without internal derangement. Oral Surg Oral Med Oral Pathol. 1992; 73:118–22. 10.1016/0030-4220(92)90168-p 1603550

[3] Kalladka M, Quek S, Heir G, Eliav E, Mupparapu M, Viswanath A. Temporomandibular joint osteoarthritis: diagnosis and long-term conservative management: a topic review. J Indian Prosthodont Soc. 2014; 14:6–15. 10.1007/s13191-013-0321-3 24604992 PMC3935038

[4] Asanuma K, Yokota S, Chosa N, Kamo M, Ibi M, Mayama H, Irié T, Satoh K, Ishisaki A. Hydrogen peroxide-induced oxidative stress promotes expression of CXCL15/Lungkine mRNA in a MEK/ERK-dependent manner in fibroblast-like synoviocytes derived from mouse temporomandibular joint. J Oral Biosci. 2023; 65:97–103. 10.1016/j.job.2022.12.002 36584898

[5] Xiong L, Bao H, Li S, Gu D, Li Y, Yin Q, Li W, Miao L, Liu C. Cerium oxide nanoparticles protect against chondrocytes and cartilage explants from oxidative stress via Nrf2/HO-1 pathway in temporomandibular joint osteoarthritis. Front Bioeng Biotechnol. 2023; 11:1076240. 10.3389/fbioe.2023.1076240 36815898 PMC9937079

[6] Miao Y, Chen Y, Xue F, Liu K, Zhu B, Gao J, Yin J, Zhang C, Li G. Contribution of ferroptosis and GPX4’s dual functions to osteoarthritis progression. EBioMedicine. 2022; 76:103847. 10.1016/j.ebiom.2022.103847 35101656 PMC8822178

[7] Hosseinzadeh A, Kamrava SK, Joghataei MT, Darabi R, Shakeri-Zadeh A, Shahriari M, Reiter RJ, Ghaznavi H, Mehrzadi S. Apoptosis signaling pathways in osteoarthritis and possible protective role of melatonin. J Pineal Res. 2016; 61:411–25. 10.1111/jpi.12362 27555371

[8] Matsuzaki T, Alvarez-Garcia O, Mokuda S, Nagira K, Olmer M, Gamini R, Miyata K, Akasaki Y, Su AI, Asahara H, Lotz MK. FoxO transcription factors modulate autophagy and proteoglycan 4 in cartilage homeostasis and osteoarthritis. Sci Transl Med. 2018; 10:eaan0746. 10.1126/scitranslmed.aan0746 29444976 PMC6204214

[9] Yao X, Sun K, Yu S, Luo J, Guo J, Lin J, Wang G, Guo Z, Ye Y, Guo F. Chondrocyte ferroptosis contribute to the progression of osteoarthritis. J Orthop Translat. 2020; 27:33–43. 10.1016/j.jot.2020.09.006 33376672 PMC7750492

[10] van Vulpen LF, Roosendaal G, van Asbeck BS, Mastbergen SC, Lafeber FP, Schutgens RE. The detrimental effects of iron on the joint: a comparison between haemochromatosis and haemophilia. J Clin Pathol. 2015; 68:592–600. 10.1136/jclinpath-2015-202967 25897098

[11] Nieuwenhuizen L, Schutgens RE, van Asbeck BS, Wenting MJ, van Veghel K, Roosendaal G, Biesma DH, Lafeber FP. Identification and expression of iron regulators in human synovium: evidence for upregulation in haemophilic arthropathy compared to rheumatoid arthritis, osteoarthritis, and healthy controls. Haemophilia. 2013; 19:e218–27. 10.1111/hae.12208 23777533

[12] Yao X, Zhang J, Jing X, Ye Y, Guo J, Sun K, Guo F. Fibroblast growth factor 18 exerts anti-osteoarthritic effects through PI3K-AKT signaling and mitochondrial fusion and fission. Pharmacol Res. 2019; 139:314–24. 10.1016/j.phrs.2018.09.026 30273654

[13] Qiu L, Luo Y, Chen X. Quercetin attenuates mitochondrial dysfunction and biogenesis via upregulated AMPK/SIRT1 signaling pathway in OA rats. Biomed Pharmacother. 2018; 103:1585–91. 10.1016/j.biopha.2018.05.003 29864946

[14] Chen X, Yang J, Shen M, Chen Y, Yu Q, Xie J. Structure, function and advance application of microwave-treated polysaccharide: A review, Trends in Food Science and Technology. 2022; 123:198–209. 10.1016/j.tifs.2022.03.016

[15] Abusarah J, Bentz M, Benabdoune H, Rondon PE, Shi Q, Fernandes JC, Fahmi H, Benderdour M. An overview of the role of lipid peroxidation-derived 4-hydroxynonenal in osteoarthritis. Inflamm Res. 2017; 66:637–51. 10.1007/s00011-017-1044-4 28447122

[16] Dixon SJ, Lemberg KM, Lamprecht MR, Skouta R, Zaitsev EM, Gleason CE, Patel DN, Bauer AJ, Cantley AM, Yang WS, Morrison B 3rd, Stockwell BR. Ferroptosis: an iron-dependent form of nonapoptotic cell death. Cell. 2012; 149:1060–72. 10.1016/j.cell.2012.03.042 22632970 PMC3367386

[17] Stockwell BR, Friedmann Angeli JP, Bayir H, Bush AI, Conrad M, Dixon SJ, Fulda S, Gascón S, Hatzios SK, Kagan VE, Noel K, Jiang X, Linkermann A, et al. Ferroptosis: A Regulated Cell Death Nexus Linking Metabolism, Redox Biology, and Disease. Cell. 2017; 171:273–85. 10.1016/j.cell.2017.09.021 28985560 PMC5685180

[18] Rannou F, Pelletier JP, Martel-Pelletier J. Efficacy and safety of topical NSAIDs in the management of osteoarthritis: Evidence from real-life setting trials and surveys. Semin Arthritis Rheum. 2016; 45:S18–21. 10.1016/j.semarthrit.2015.11.007 26806189

[19] Lefkowith JB. Cyclooxygenase-2 specificity and its clinical implications. Am J Med. 1999; 106:43S–50S. 10.1016/s0002-9343(99)00116-3 10390127

[20] Guan HH, Huang YH, Lin ES, Chen CJ, Huang CY. Plumbagin, a Natural Product with Potent Anticancer Activities, Binds to and Inhibits Dihydroorotase, a Key Enzyme in Pyrimidine Biosynthesis. Int J Mol Sci. 2021; 22:6861. 10.3390/ijms22136861 34202294 PMC8267945

[21] Roy A. Plumbagin: A Potential Anti-cancer Compound. Mini Rev Med Chem. 2021; 21:731–7. 10.2174/1389557520666201116144421 33200707

[22] Pai SA, Munshi RP, Panchal FH, Gaur IS, Mestry SN, Gursahani MS, Juvekar AR. Plumbagin reduces obesity and nonalcoholic fatty liver disease induced by fructose in rats through regulation of lipid metabolism, inflammation and oxidative stress. Biomed Pharmacother. 2019; 111:686–94. 10.1016/j.biopha.2018.12.139 30611993

[23] Zheng W, Tao Z, Chen C, Zhang C, Zhang H, Ying X, Chen H. Plumbagin Prevents IL-1β-Induced Inflammatory Response in Human Osteoarthritis Chondrocytes and Prevents the Progression of Osteoarthritis in Mice. Inflammation. 2017; 40:849–60. 10.1007/s10753-017-0530-8 28168658

[24] Shu C, Chen J, Lv M, Xi Y, Zheng J, Xu X. Plumbagin relieves rheumatoid arthritis through nuclear factor kappa-B (NF-κB) pathway. Bioengineered. 2022; 13:13632–42. 10.1080/21655979.2022.2081756 35653787 PMC9276045

[25] Chen T, Zhou R, Chen Y, Fu W, Wei X, Ma G, Hu W, Lu C. Curcumin ameliorates IL-1β-induced apoptosis by activating autophagy and inhibiting the NF-κB signaling pathway in rat primary articular chondrocytes. Cell Biol Int. 2021; 45:976–88. 10.1002/cbin.11541 33377585

[26] Wang YL, Zhang J, Zhang M, Lu L, Wang X, Guo M, Zhang X, Wang MQ. Cartilage degradation in temporomandibular joint induced by unilateral anterior crossbite prosthesis. Oral Dis. 2014; 20:301–6. 10.1111/odi.12112 23614573

[27] Chen X, Wang X, Shen M, Chen Y, Yu Q, Yang J, Xie J. Combined RNA-seq and molecular biology technology revealed the protective effect of Cyclocarya paliurus polysaccharide on H2O2-induced oxidative damage in L02 cells thought regulating mitochondrial function, oxidative stress and PI3K/Akt and MAPK signaling pathways. Food Res Int. 2022; 155:111080. 10.1016/j.foodres.2022.111080 35400456

[28] de Souza RF, Lovato da Silva CH, Nasser M, Fedorowicz Z, Al-Muharraqi MA. Interventions for the management of temporomandibular joint osteoarthritis. Cochrane Database Syst Rev. 2012; 2012:CD007261. 10.1002/14651858.CD007261.pub2 22513948 PMC6513203

[29] Tong L, Yu H, Huang X, Shen J, Xiao G, Chen L, Wang H, Xing L, Chen D. Current understanding of osteoarthritis pathogenesis and relevant new approaches. Bone Res. 2022; 10:60. 10.1038/s41413-022-00226-9 36127328 PMC9489702

[30] Yuan W, Wu Y, Huang M, Zhou X, Liu J, Yi Y, Wang J, Liu J. A new frontier in temporomandibular joint osteoarthritis treatment: Exosome-based therapeutic strategy. Front Bioeng Biotechnol. 2022; 10:1074536. 10.3389/fbioe.2022.1074536 36507254 PMC9732036

[31] Zhang Z, Yuan L, Liu Y, Wang R, Zhang Y, Yang Y, Wei H, Ma J. Integrated Cascade Nanozyme Remodels Chondrocyte Inflammatory Microenvironment in Temporomandibular Joint Osteoarthritis via Inhibiting ROS-NF-κB and MAPK Pathways. Adv Healthc Mater. 2023; 12:e2203195. 10.1002/adhm.202203195 36738173

[32] Ren JX, Li C, Yan XL, Qu Y, Yang Y, Guo ZN. Crosstalk between Oxidative Stress and Ferroptosis/Oxytosis in Ischemic Stroke: Possible Targets and Molecular Mechanisms. Oxid Med Cell Longev. 2021; 2021:6643382. 10.1155/2021/6643382 34055196 PMC8133868

[33] Guo YX, Liu L, Yan DZ, Guo JP. Plumbagin prevents osteoarthritis in human chondrocytes through Nrf-2 activation. Mol Med Rep. 2017; 15:2333–8. 10.3892/mmr.2017.6234 28259976

[34] Chu H, Yu H, Ren D, Zhu K, Huang H. Plumbagin exerts protective effects in nucleus pulposus cells by attenuating hydrogen peroxide-induced oxidative stress, inflammation and apoptosis through NF-κB and Nrf-2. Int J Mol Med. 2016; 37:1669–76. 10.3892/ijmm.2016.2564 27082014

[35] Mapp PI, Walsh DA. Mechanisms and targets of angiogenesis and nerve growth in osteoarthritis. Nat Rev Rheumatol. 2012; 8:390–8. 10.1038/nrrheum.2012.80 22641138

[36] Hu Y, Chen X, Wang S, Jing Y, Su J. Subchondral bone microenvironment in osteoarthritis and pain. Bone Res. 2021; 9:20. 10.1038/s41413-021-00147-z 33731688 PMC7969608

[37] Cui Z, Crane J, Xie H, Jin X, Zhen G, Li C, Xie L, Wang L, Bian Q, Qiu T, Wan M, Xie M, Ding S, et al. Halofuginone attenuates osteoarthritis by inhibition of TGF-β activity and H-type vessel formation in subchondral bone. Ann Rheum Dis. 2016; 75:1714–21. 10.1136/annrheumdis-2015-207923 26470720 PMC5013081

[38] Lai L, Liu J, Zhai D, Lin Q, He L, Dong Y, Zhang J, Lu B, Chen Y, Yi Z, Liu M. Plumbagin inhibits tumour angiogenesis and tumour growth through the Ras signalling pathway following activation of the VEGF receptor-2. Br J Pharmacol. 2012; 165:1084–96. 10.1111/j.1476-5381.2011.01532.x 21658027 PMC3346245

[39] Sultanli S, Ghumnani S, Ashma R, Kubatzky KF. Plumbagin, a Biomolecule with (Anti)Osteoclastic Properties. Int J Mol Sci. 2021; 22:2779. 10.3390/ijms22052779 33803472 PMC7967158

[40] Behl T, Rana T, Alotaibi GH, Shamsuzzaman M, Naqvi M, Sehgal A, Singh S, Sharma N, Almoshari Y, Abdellatif AAH, Iqbal MS, Bhatia S, Al-Harrasi A, Bungau S. Polyphenols inhibiting MAPK signalling pathway mediated oxidative stress and inflammation in depression. Biomed Pharmacother. 2022; 146:112545. 10.1016/j.biopha.2021.112545 34922112

[41] Fang JY, Richardson BC. The MAPK signalling pathways and colorectal cancer. Lancet Oncol. 2005; 6:322–7. 10.1016/S1470-2045(05)70168-6 15863380

[42] Behl T, Upadhyay T, Singh S, Chigurupati S, Alsubayiel AM, Mani V, Vargas-De-La-Cruz C, Uivarosan D, Bustea C, Sava C, Stoicescu M, Radu AF, Bungau SG. Polyphenols Targeting MAPK Mediated Oxidative Stress and Inflammation in Rheumatoid Arthritis. Molecules. 2021; 26:6570. 10.3390/molecules26216570 34770980 PMC8588006

[43] Yu Y, Xie Y, Cao L, Yang L, Yang M, Lotze MT, Zeh HJ, Kang R, Tang D. The ferroptosis inducer erastin enhances sensitivity of acute myeloid leukemia cells to chemotherapeutic agents. Mol Cell Oncol. 2015; 2:e1054549. 10.1080/23723556.2015.1054549 27308510 PMC4905356

[44] Poursaitidis I, Wang X, Crighton T, Labuschagne C, Mason D, Cramer SL, Triplett K, Roy R, Pardo OE, Seckl MJ, Rowlinson SW, Stone E, Lamb RF. Oncogene-Selective Sensitivity to Synchronous Cell Death following Modulation of the Amino Acid Nutrient Cystine. Cell Rep. 2017; 18:2547–56. 10.1016/j.celrep.2017.02.054 28297659 PMC5368412

[45] Ma N, Teng X, Zheng Q, Chen P. The regulatory mechanism of p38/MAPK in the chondrogenic differentiation from bone marrow mesenchymal stem cells. J Orthop Surg Res. 2019; 14:434. 10.1186/s13018-019-1505-2 31831024 PMC6909593

[46] Li Z, Dai A, Yang M, Chen S, Deng Z, Li L. p38MAPK Signaling Pathway in Osteoarthritis: Pathological and Therapeutic Aspects. J Inflamm Res. 2022; 15:723–34. 10.2147/JIR.S348491 35140502 PMC8820459

[47] Shan W, Cheng C, Huang W, Ding Z, Luo S, Cui G, Lu W, Liu F, Xu J, He W, Yin Z. Angiopoietin-like 2 upregulation promotes human chondrocyte injury via NF-κB and p38/MAPK signaling pathway. J Bone Miner Metab. 2019; 37:976–86. 10.1007/s00774-019-01016-w 31214838

[48] Guo YN, Cui SJ, Tian YJ, Zhao NR, Zhang YD, Gan YH, Zhou YH, Wang XD. Chondrocyte apoptosis in temporomandibular joint osteoarthritis promotes bone resorption by enhancing chemotaxis of osteoclast precursors. Osteoarthritis Cartilage. 2022; 30:1140–53. 10.1016/j.joca.2022.04.002 35513247

[49] Zheng XY, Mao CY, Qiao H, Zhang X, Yu L, Wang TY, Lu EY. Plumbagin suppresses chronic periodontitis in rats via down-regulation of TNF-α, IL-1β and IL-6 expression. Acta Pharmacol Sin. 2017; 38:1150–60. 10.1038/aps.2017.19 28552911 PMC5547554

