# Supplementary material for: Plumbagin alleviates temporomandibular joint osteoarthritis progression by inhibiting chondrocyte ferroptosis via the MAPK signaling pathways
Source: Aging (Albany NY). 2023 Nov 29;15(22):13452–70. doi: 10.18632/aging.205253 (PMC10713407; doi:10.18632/aging.205253)
Supplement: Supplementary Figures [file aging-15-205253-s001.pdf]

SUPPLEMENTARY FIGURES

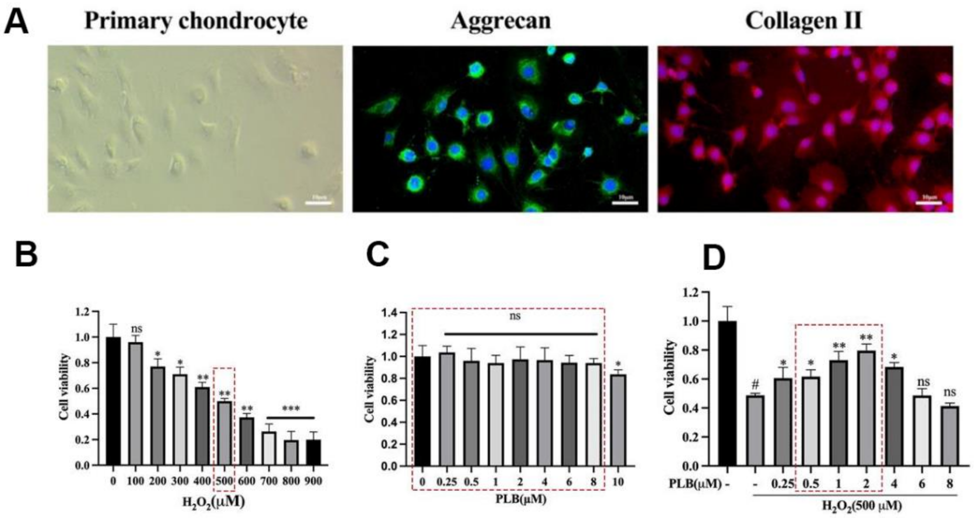

Supplementary Figure 1. PLB inhibited chondrocytes death caused by H_2O_2. (**A**) Verification of rat primary chondrocytes with cell morphology, aggrecan and collagen II. Scale bars, 10 μm. (**B**–**D**) Chondrocyte's viability determined by CCK-8 assay. (**B**) Chondrocytes were incubated with various concentrations H_2O_2 (0, 100, 200, 300, 400, 500 600, 700, 800 and 900 μM) and 12 h. n = 6. (**C**) Chondrocytes were incubated with PLB various concentrations (0, 0.25, 0.5, 1, 2, 4, 6, 8 and 10 μM) and 12 h. n = 6. (**D**) Chondrocytes were incubated with PLB various concentrations (0, 0.25, 0.5, 1, 2, 4, 6 and 8 μM) for 12 h than 500 μM H_2O_2 for 12 h. n = 6. (All quantified data are presented as mean ± SEM; ns, not significant, #P < 0.05, *P < 0.05, **P < 0.01, ***P < 0.001 by one-way ANOVA and the Tukey-Kramer test. #: CON vs H_2O_2, *: H_2O_2 vs PLB+ H_2O_2).

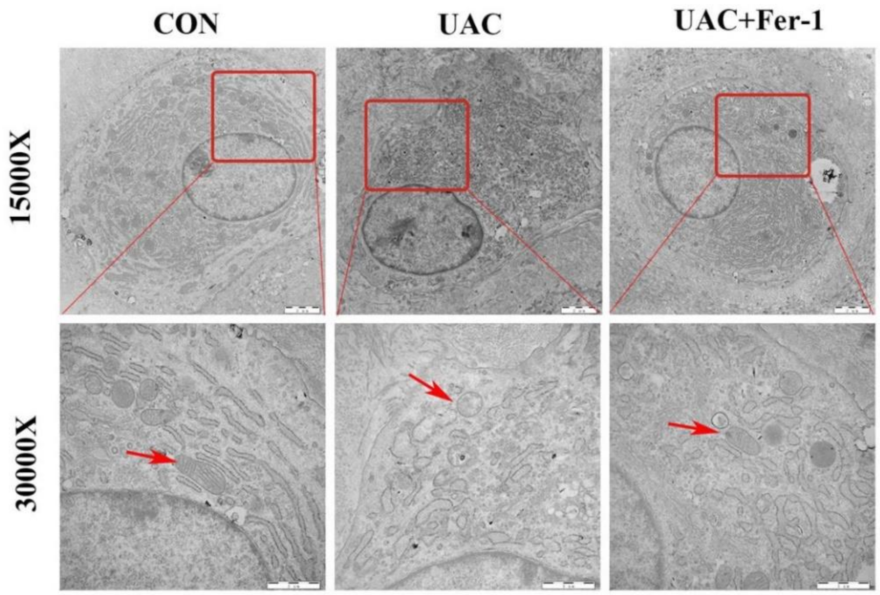

Supplementary Figure 2. PLB recovered mitochondrial membrane morphology of chondrocytes induced by UAC. CON: healthy mitochondria exhibit an elliptical or elongated shape, with a bilayer membrane structure. UAC: chondrocytes stimulated under UAC, mitochondria tend to adopt a more spherical and bloated morphology, leading to an increase in volume or even outer membrane rupture. UAC+Fer-1: the morphological alterations caused by UAC can be prevented by the administration of Fer-1.

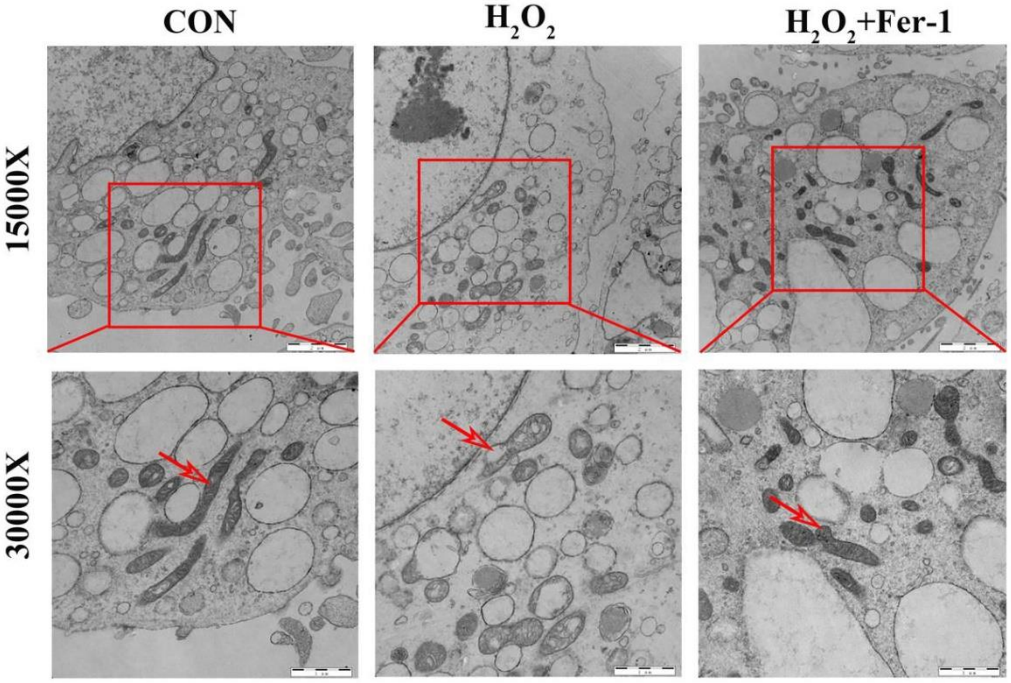

Supplementary Figure 3. PLB recovered mitochondrial membrane morphology of chondrocytes induced by H_2O_2. CON: healthy mitochondria exhibit an elliptical or elongated shape, with a bilayer membrane structure. H_2O_2: chondrocytes stimulated with H_2O_2 (500 μM), mitochondria tend to adopt a more spherical and bloated morphology, leading to an increase in volume or even outer membrane rupture. H_2O_2+Fer-1: the morphological alterations caused by H_2O_2 can be prevented by the administration of Fer-1.

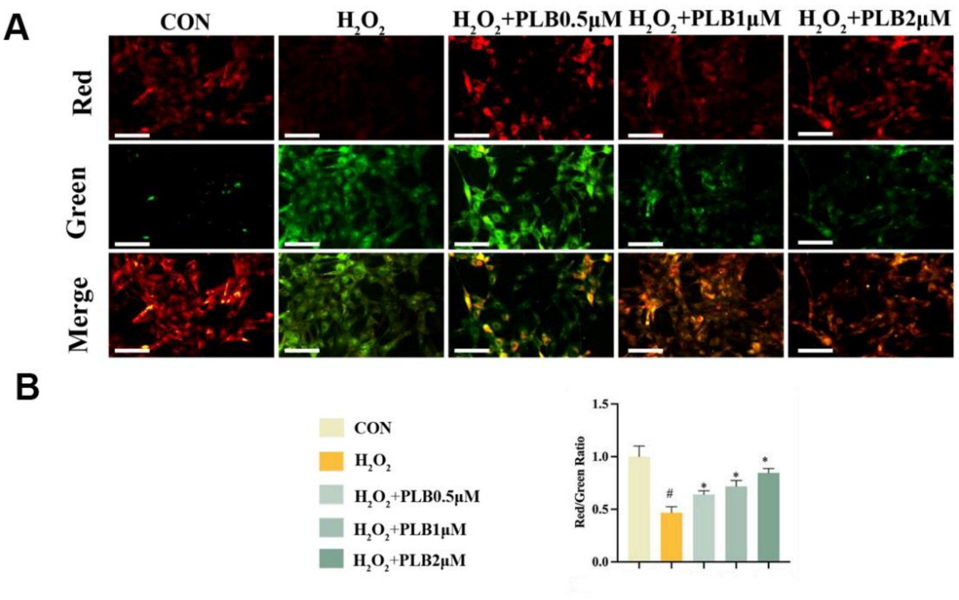

Supplementary Figure 4. PLB recovered mitochondrial membrane potential of chondrocytes induced by H_2O_2. (**A**) JC-1 measurement for mitochondrial membrane potential levels of chondrocytes after various treatments. Red indicates high membrane potential, Green indicates low membrane potential, and Merge indicates the combination of the two. Scale bars, 100 μm. (**B**) Quantitative analysis for mitochondrial membrane potential level of chondrocytes after various treatments. n = 3. (All quantified data are presented as mean ± SEM; #$P < 0.05$ *$P < 0.05$ by one-way ANOVA and the Tukey-Kramer test. #: CON vs H_2O_2, *: H_2O_2 vs PLB+ H_2O_2).
