# Supplementary material for: Plumbagin alleviates temporomandibular joint osteoarthritis progression by inhibiting chondrocyte ferroptosis via the MAPK signaling pathways
Source: Aging (Albany NY). 2023 Nov 29;15(22):13452–70. doi: 10.18632/aging.205253 (PMC10713407; doi:10.18632/aging.205253)
Supplement: Supplementary Table 1 [file aging-15-205253-s002.pdf]

SUPPLEMENTARY TABLE

Supplementary Table 1. Primer sequences.

Genes	Forward (5'-3')	Reverse (5'-3')
Col2α1	GTGTCAAGGGTCACAGAGGTTAC	CGCTCTCACCCTTCACACCT
ACAN	AGTGACCCATCTGCTTACCCTG	CTGCATCTATGTCGGAGGTAGTG
MMP-13	ATGTGACACCTCTGAATTTTACCAG	CATGGGCAGCAACAATAAATAAG
Adamts-5	CCTGAAGGAGCAACCCACAT	GCTTTGGGTAGGCCTTGTCT
GPX-4	AAAGTCCTAGGAAGCGCCCA	GTGGGCATCGTCCCCATTTA
SLC7A11	AGGCGGTAGCATAAATAGGAGC	GTGACAGTACTCCACAGGCA
ACSL4	CCTCCGGGCTCGTCTTTTC	GCTGTGAATCTCAAGCCCCT
PTGS2	AGGGCCCTACCAAGATGCTA	AATTTCTCTGAGGCACGGCA
β-actin	CACCCGCGAGTACAACCTTC	GTACATGGCTGGGGTGTTGA
